# The use of artificial intelligence based chat bots in ophthalmology triage

**DOI:** 10.1038/s41433-024-03488-1

**Published:** 2024-11-26

**Authors:** Daniel David, Ofira Zloto, Gabriel Katz, Ruth Huna-Baron, Vicktoria Vishnevskia-Dai, Sharon Armarnik, Noa Avni Zauberman, Elinor Megiddo Barnir, Reut Singer, Avner Hostovsky, Eyal Klang

**Affiliations:** 1 Faculty of Medicine, Tel Aviv University, Tel Aviv, Israel; 2 Goldschleger Eye Institute, Sheba Medical Center, Tel Hashomer, Israel; 3 Division of Data-Driven and Digital Medicine (D3M), Icahn School of Medicine at Mount Sinai, New York, NY USA

**Keywords:** Epidemiology, Education

## Abstract

**Purpose:**

To evaluate AI-based chat bots ability to accurately answer common patient’s questions in the field of ophthalmology.

**Methods:**

An experienced ophthalmologist curated a set of 20 representative questions and responses were sought from two AI generative models: OpenAI’s ChatGPT and Google’s Bard (Gemini Pro). Eight expert ophthalmologists from different sub-specialties assessed each response, blinded to the source, and ranked them by three metrics—accuracy, comprehensiveness, and clarity, on a 1–5 scale.

**Results:**

For accuracy, ChatGPT scored a median of 4.0, whereas Bard scored a median of 3.0. In terms of comprehensiveness, ChatGPT achieved a median score of 4.5, compared to Bard which scored a median of 3.0. Regarding clarity, ChatGPT maintained a higher score with a median of 5.0, compared to Bard’s median score of 4.0. All comparisons were statistically significant (*p* < 0.001).

**Conclusion:**

AI-based chat bots can provide relatively accurate and clear responses for addressing common ophthalmological inquiries. ChatGPT surpassed Bard in all measured metrics. While these AI models exhibit promise, further research is indicated to improve their performance and allow them to be used as a reliable medical tool.

## Introduction

In recent years, artificial intelligence (AI) has been increasingly deployed in clinical practice. From image analysis in radiology [1] to natural language processing (NLP) for electronic health records [2], AI technologies are optimizing healthcare workflows, improving diagnostic accuracy, and enabling the customization of treatment strategies. Machine learning algorithms are aiding in the early detection of diseases [3, 4] and provide healthcare professionals with data-driven insights.

The development of AI-based chat bots, such as OpenAI’s ChatGPT (Generative Pre-trained Transformer) and Google’s Bard (now Gemini Pro), represents another significant advancement in the field of AI. These chat bots are built upon sophisticated NLP models. They are pre-trained on vast amounts of text data, allowing them to understand and generate human-like text responses [5].

Although only recently introduced, in the medical context, AI-based chat bots already have diverse applications. They serve as popular and accessible resources for answering medical questions, offering information on symptoms, treatments, and general health advice [6]. Patients can now inquire about a wide range of medical topics, without the need for immediate medical consultation [7].

However, it is important to note that while AI-based chat bots in medicine offer many advantages, their responses are generated based on the data they have been trained on and may lack the personalized context that a healthcare provider can offer. Therefore, their use is best suited for providing general information and initial guidance, complementing the work of medical professionals [8].

While the implementation of chat bots in various medical specialties has been met with enthusiasm, it is vital to critically assess their accuracy and reliability in addressing patient inquiries.

In this paper, we aim to evaluate the performance of AI-based chat bots, specifically ChatGPT and Bard, in their ability to accurately answer common patient’s questions in the field of ophthalmology.

## Methods

### Question selection

An experienced ophthalmologist curated a set of 20 representative questions from a pool of 100 consecutive patient inquiries. These questions were deemed the most common and clinically relevant.

### Evaluation of AI models

Responses were sought from two AI generative models on the 16/07/2023: OpenAI’s ChatGPT version GPT-3.5 and Google’s Bard. Using the web interface for both models, the 20 selected questions were prompted verbatim. Responses generated by each model were collected without alterations. Of note, since February 2024 Google changes the name from Bard to Gemini.

### Expert review and scoring

Eight expert ophthalmologists, blinded to the type of AI model, assessed each response. The evaluations revolved around three metrics: Accuracy (alignment of the answer with established clinical knowledge), Comprehensiveness (the depth of the answer), and Clarity (the ease of understanding of the response). Each metric was scored on a scale from 1 to 5. The evaluating ophthalmologists were instructed to score every metric of each answer as follows: 1 = indicates a low-quality answer: not aligning with clinical knowledge, incomprehensive and unclear; 2 = moderate-low quality answer: mild clinical relevance, poor comprehensiveness and clarity; 3 = moderate-quality answer: some clinical relevance, acceptable comprehensiveness and clarity; 4 = moderate-good quality answer: most of it is clinically relevant, comprehensive and clear; 5 = good-quality answer: aligned with clinical knowledge, very comprehensive and clear.

### Statistical analysis

Statistical analyses were executed using Python version 3.10.13.

Initial descriptive statistics provided an overview of the ratings—median and interquartile range (IQR) for each metric across both AI models.

To determine if significant differences existed between the ratings of ChatGPT and Bard for each metric, the Mann–Whitney U test was employed. A significance threshold was set at *p* < 0.05.

The consensus among raters was also assessed by quantifying the percentage of ophthalmologists’ ratings which landed within ±1 point on the median for a given metric.

Lastly, an error analysis was conducted, serving as a qualitative review of questions that revealed inconsistent scores or distinct performance disparities between the models.

## Results

The comparative analysis between ChatGPT and Bard revealed distinct differences in performance across various metrics. For accuracy, ChatGPT scored a median of 4.0, whereas Bard scored a median of 3.0. In terms of comprehensiveness, ChatGPT achieved a median score of 4.5, significantly outperforming Bard, which scored a median of 3.0. Regarding clarity, ChatGPT maintained a higher score with a median of 5.0, compared to Bard’s median score of 4.0.

**Fig. 1:**
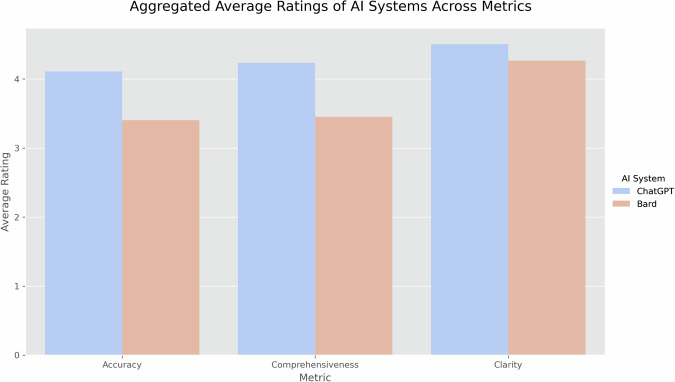
Boxplot comparing ChatGPT and Bard ratings. This figure showcases representation of the overall median ratings received by ChatGPT and Bard for the metrics of Accuracy, Comprehensiveness, and Clarity.

**Fig. 2:**
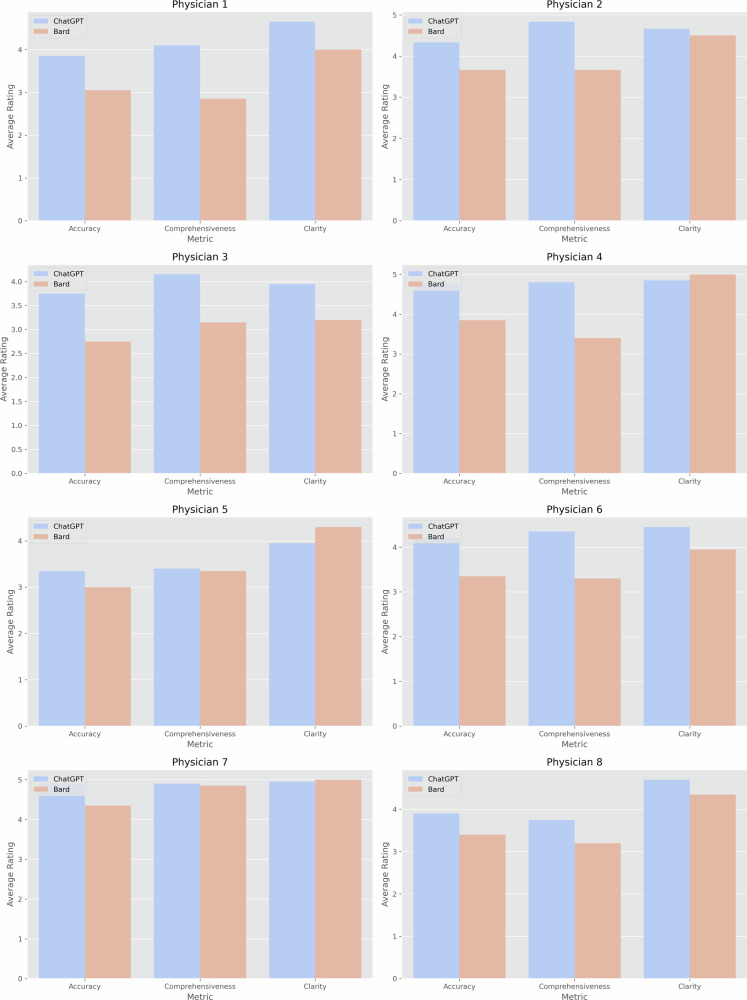
Individual physician ratings for ChatGPT and Bard. A breakdown of ratings assigned by each of the eight physicians to ChatGPT and Bard across the three metrics.

Figure 1 presents a comparison of the average ratings for each metric between ChatGPT and Bard. Figure 2 provides a similar comparison but for each separate physician. All comparisons were statistically significant (*p* < 0.001). These results indicate a consistently higher performance by ChatGPT across all assessed metrics.

### Consensus measure

The average consensus among ophthalmologists for ChatGPT was 82.5% for both Accuracy and Comprehensiveness, and 83.75% for Clarity. Bard’s ratings showed lower agreement, with an average consensus of 76.9% for Accuracy, 74.4% for Comprehensiveness, and 83.8% for Clarity (Table 1).

**Table 1:** Consensus measure results for ChatGPT and Bard across metrics.

Metric	Average consensus (%)
ChatGPT accuracy	82.5
ChatGPT comprehensiveness	82.5
ChatGPT clarity	83.8
Bard accuracy	76.9
Bard comprehensiveness	74.4
Bard clarity	83.8

The table presents the percentage of physicians’ ratings who were in close agreement (within ±1 point on the scale).

### Error analysis

Upon examining specific questions (supplementary data 1 and 2), clear differences between ChatGPT and Bard were observed. For the topic of “red eye with discomfort and discharge” (question 1), ChatGPT generally received higher scores for accuracy than Bard. While the first chat bot provides an extensive differential diagnosis and treatment options, Bard’s answer focuses only on the different causes of conjunctivitis, ignoring other vision-threatening conditions. Bard’s answer to the “causes and treatment of double vision” (question 10) lacked accuracy and comprehensiveness, addressing only binocular diplopia, omitting important monocular causes, such as cataract. ChatGPT’s response, on the other hand, includes monocular causes, though no categorization is noted.

Both models provided clear answers about “the best timing for cataract surgery” (question 7), denoting the different considerations for procedure scheduling. Yet none of the models addressed the concept of preforming surgery for better visualization of the posterior segment in cases such as choroidal melanomas.

An example for an inaccurate, yet comprehensive answer is ChatGPT’s response for “what is the treatment for a retinal tear?” (question 4). The chat bot elaborates on the different treatments yet there is no clear distinction between retinal tear and retinal detachment, which might lead patients with small low-risk tears to believe they’re in need for a surgery. Bard’s answer, on the other hand, focuses on the differences between retinal tear and retinal detachment but the treatment options is not as elaborated as in the first AI model answer.

Generally, wrong answers had minor errors, for example Chat-GPT’s answer to the question “what are the warning signs and symptoms for retinal detachment?” (question 8) included pain as one of the symptoms when in fact it is a painless condition. Notwithstanding, some answers had major errors that can lead to inaccurate diagnosis, like Chat-GPT’s answer to question 15—“my right eye looks smaller than my left one, what is the cause and how can I treat it?”—addressing only right eye ptosis and omitting a wide differential diagnosis including left eye proptosis.

## Discussion

AI-based chat bots have recently emerged as accessible resources for providing medical information to patients [5]. These chat bots are built on NLP and machine learning, offering human-like text responses. As these chat bots become increasingly popular, it is important to evaluate their accuracy, to assist in both patient and physician decision making.

In contrast with their wide use, evidence-based data evaluating the chat bot’s scientific accuracy in answering patients’ questions is infrequent. Lahat et al. [9]. evaluated the performance of ChatGPT in answering patient’s questions in the field of gastroenterology. Their results showed that ChatGPT was able to provide accurate answers to patient’s questions in some, but not all, cases. The most accurate answers were given to questions regarding the treatment of specific medical conditions, while answers describing the disease’s symptoms were the least accurate.

Our work focuses on evaluating the accuracy, comprehensiveness, and clarity of AI-based chat bots in addressing common patient queries within the field of ophthalmology.

Our results show that both ChatGPT and Bard can provide good, clear answers to patient’s questions in clinical ophthalmology. This is in accordance with previous studies which found that chat bots are a promising diagnostic adjunct in ophthalmology but still cannot be a replacement for professional ophthalmic evaluation [10–12].

In this current study ChatGPT exhibited higher median ratings for Accuracy (4.0 vs. 3.0), Comprehensiveness (4.5 vs. 3.0), and Clarity (5.0 vs. 4.0) in the expert’s evaluations compared to Bard. These disparities signify a substantial, statistically significant, variance in the models’ capabilities to deliver accurate, comprehensive, and lucid responses to ophthalmology queries, and puts ChatGPT in a relative advantage in these aspects.

Other recent studies that compared between Bard and ChatGPT also found that the answers that were given by ChatGPT were more accurate [13, 14].

In our study, eight consultants form different ophthalmology subspecialties have compared the answers. This number of experts and their diversity is relatively high compared to previous studies [13, 14].

Our study is not without any limitations, although blinded to the specific AI model, expert’s evaluations are inherently biased and effected by their own clinical knowledge and experience. Moreover, conclusions are based on specific questions and might differ if the questions were drafted in a different manner. As these AI models are constantly improving, generating a question today will not necessarily yield the same answer as when we first used these models and likewise when used in the future. Other AI-based chat bots were not evaluated in this paper and their accuracy in answering questions in clinical ophthalmology remains to be studied. Moreover, we used the web interface to query the models, thus we did not evaluate hyper-parameter tuning, nor other advanced techniques such as retrieval augmented generation or fine-tuning. Also, we did not explore prompt engineering, rather we prompted using a simple straight-forward prompt. However, using a web-interface replicates the common interaction of patients with chat bots, which we wanted to simulate in our study.

In conclusion, our study highlights the potential utility of chat bots, especially ChatGPT, as supplementary resources for addressing common patient ophthalmology inquiries. While these AI models exhibit promise, the disparities in their performance emphasize the need for ongoing refinement and optimization to align more closely with expert-level responses. Future research should focus on enhancing the comprehensiveness, accuracy, and clarity of AI-driven responses to meet the demands of clinical ophthalmology practice.

## Summary

### What was known before

- Many patients use chat bots for medical inquiries. Artificial intelligence is increasingly deployed in clinical practice.

### What this study adds

- AI-based chat bots can provide relatively accurate and clear responses for addressing common ophthalmological inquiries. ChatGPT surpassed Bard in all measured metrics.

## Supplementary information

Q and A ChatGPT

Q and A BARD

Supplementary Materials

## Data Availability

All research data including the chat bot’s full questions and answers are elaborated in the supplementary files (supplementary data 1 and 2).
