# Supplementary material for: The use of artificial intelligence based chat bots in ophthalmology triage
Source: Eye (Lond). 2024 Nov 26;39(4):785–9. doi: 10.1038/s41433-024-03488-1 (PMC11885819; doi:10.1038/s41433-024-03488-1)
Supplement: Supplementary file 1 — Q and A ChatGPT [file 41433_2024_3488_MOESM1_ESM.docx]

**Chat GPT – Answers to Common Questions in Ophthalmology**

**Q1:**

I have a red eye with discomfort and discharge, what is the diagnosis and treatment?

**A1:**

I am not a medical professional, but I can offer some general information. If you have a red eye with discomfort and discharge, it could be caused by various conditions, and it's important to consult a healthcare professional or an eye doctor for a proper diagnosis and treatment plan. Some possible causes include:

1. Conjunctivitis (Pink Eye): It is a common condition that can be caused by viruses, bacteria, allergies, or irritants. It can lead to redness, itching, discharge, and discomfort.

2. Eye Allergies: Allergens such as pollen, pet dander, or dust can cause redness, itching, and discharge in the eyes.

3. Foreign Object: A foreign object in the eye can cause irritation, redness, and discomfort.

4. Eye Infections: Various infections, such as bacterial or viral infections, can affect the eye and cause redness and discharge.

5. Dry Eyes: Insufficient tear production can lead to dryness, discomfort, and possibly redness.

6. Blepharitis: Inflammation of the eyelids can cause redness, discharge, and a gritty sensation in the eyes.

7. Corneal Abrasion: A scratch on the surface of the cornea can cause discomfort, redness, and sensitivity to light.

Treatment will depend on the underlying cause of your symptoms. It may include:

- Antibiotic eye drops or ointments for bacterial infections.

- Antiviral medication for viral infections.

- Anti-allergy eye drops or oral antihistamines for eye allergies.

- Lubricating eye drops for dry eyes.

- Warm compresses and eyelid hygiene for blepharitis.

- Removing any foreign objects from the eye.

- Avoiding irritants or allergens that may be causing the issue.

Again, it's crucial to seek advice from a healthcare professional to determine the specific cause of your symptoms and to receive appropriate treatment. They can provide a proper diagnosis and personalized recommendations based on your medical history and examination.

**Q2:**

I have a painful, localized swelling of the eyelid, what is the diagnosis and treatment?

**A2:**

I am not a medical professional, but I can provide some general information. A painful, localized swelling of the eyelid could be caused by several conditions, and it's essential to consult with a healthcare professional or an eye doctor for an accurate diagnosis and appropriate treatment. Some possible causes of eyelid swelling include:

1. Stye (Hordeolum): A stye is a red, painful bump on the eyelid caused by a bacterial infection in the oil glands of the eyelids.

2. Chalazion: A chalazion is a non-infectious lump that forms when an oil gland in the eyelid becomes blocked.

3. Blepharitis: Inflammation of the eyelids can cause painful swelling in the affected area.

4. Cellulitis: A bacterial infection of the skin around the eye can lead to painful swelling and redness.

5. Allergic Reaction: An allergic reaction to certain substances can cause localized swelling and discomfort.

6. Cyst: A cyst may form on or around the eyelid, causing pain and swelling.

The treatment for eyelid swelling will depend on the underlying cause. Some general measures that may help alleviate the symptoms include:

- Warm compresses: Applying a warm compress to the affected area several times a day can help reduce pain and inflammation.

- Gentle eyelid hygiene: Keeping the eyelids clean and free from debris can aid in healing.

- Avoidance of makeup and contact lenses: If the swelling is due to an infection, avoiding makeup and contact lenses can prevent further irritation.

- Over-the-counter pain relievers: Non-prescription pain relievers may help manage discomfort, but it's essential to follow the recommended dosage.

It's crucial to seek medical advice to receive an accurate diagnosis and appropriate treatment plan for your specific situation. A healthcare professional can examine your eye and provide you with the most suitable recommendations based on your medical history and the severity of the condition.

**Q3:**

I have a painless hemorrhage on the white part of my right eye, what is the diagnosis and treatment?

**A3:**

A painless hemorrhage on the white part of the eye is often referred to as a subconjunctival hemorrhage. It occurs when a small blood vessel beneath the conjunctiva (the clear membrane covering the white part of the eye) breaks, causing blood to leak into the space between the conjunctiva and the sclera (the white part of the eye). Subconjunctival hemorrhages are usually harmless and typically resolve on their own without any treatment.

The common causes of subconjunctival hemorrhages include:

1. Eye Trauma: Minor injury or trauma to the eye, such as rubbing the eye too vigorously or getting poked in the eye, can cause a blood vessel to break.

2. Straining: Intense coughing, sneezing, vomiting, or lifting heavy objects can lead to increased pressure in the blood vessels, causing them to rupture.

3. High Blood Pressure: People with hypertension (high blood pressure) may be more prone to subconjunctival hemorrhages.

4. Blood-Thinning Medications: Certain medications, such as aspirin or anticoagulants (blood thinners), can increase the likelihood of bleeding.

5. Eye Surgery: In some cases, subconjunctival hemorrhages can occur after eye surgery.

Treatment for subconjunctival hemorrhages is generally not required, as they tend to resolve on their own within a couple of weeks. However, there are a few things you can do to promote healing and prevent complications:

1. Avoid Eye Rubbing: Refrain from rubbing or putting pressure on the affected eye, as it can worsen the hemorrhage.

2. Use Artificial Tears: Lubricating eye drops can help soothe any mild irritation or dryness in the eye.

3. Monitor Blood Pressure: If you have hypertension or other medical conditions that can cause recurrent subconjunctival hemorrhages, make sure to monitor and manage your blood pressure as advised by your healthcare provider.

4. Follow Medication Instructions: If you are taking blood-thinning medications, follow your doctor's instructions carefully and inform them about any unusual bleeding.

If you notice frequent or recurrent subconjunctival hemorrhages, or if you have other concerns or symptoms related to your eyes, it's essential to consult with an eye doctor or a healthcare professional for a proper evaluation. They can determine the underlying cause and provide you with appropriate advice and treatment if necessary.

**Q4:**

What is the treatment for a retinal tear?

**A4:**

A retinal tear is a condition where the sensory layer of the retina becomes separated or detached from the underlying tissue. It is considered a serious eye condition that requires prompt medical attention. The treatment options for a retinal tear may include:

1. Laser photocoagulation: This procedure involves using a laser to create small burns around the tear site. The burns cause scarring that helps seal the tear and prevent further fluid leakage or detachment of the retina. Laser photocoagulation is often performed in an ophthalmologist's office and is typically a quick and relatively painless procedure.

2. Cryotherapy: Cryotherapy involves using freezing temperatures to create a scar around the retinal tear. Similar to laser photocoagulation, the scar tissue helps seal the tear and prevent retinal detachment. Cryotherapy is usually performed in a hospital or surgical center under local anesthesia.

3. Pneumatic retinopexy: This procedure is suitable for certain types of retinal tears. It involves injecting a small gas bubble into the eye, which then pushes against the tear, sealing it against the wall of the eye. The patient may be positioned face-down for a period of time to allow the gas bubble to stay in contact with the tear. Pneumatic retinopexy is often combined with laser photocoagulation or cryotherapy.

4. Scleral buckle surgery: Scleral buckle surgery involves placing a silicone band or buckle around the eye to provide support and counteract the forces pulling on the retina. This helps bring the detached retina back into its proper position against the underlying tissue. Scleral buckle surgery is typically performed in a hospital under general anesthesia.

5. Vitrectomy: In more complex cases or when other treatments are not sufficient, vitrectomy surgery may be performed. This involves removing the vitreous gel inside the eye and replacing it with a clear solution. During the procedure, the retinal tear is identified and treated, and the vitreous gel is replaced to relieve traction on the retina.

The specific treatment approach for a retinal tear will depend on factors such as the location, size, and complexity of the tear, as well as the overall health of the eye. It's important to consult an ophthalmologist or a retina specialist promptly if you suspect a retinal tear to prevent the risk of retinal detachment and preserve vision.

**Q5:**

What is the treatment for blepharitis?

**A5:**

Blepharitis is a common condition characterized by inflammation of the eyelids, typically at the base of the eyelashes. The treatment for blepharitis focuses on managing the symptoms, improving eyelid hygiene, and reducing inflammation. The specific treatment approach may vary depending on the severity and underlying cause of blepharitis. Here are some common treatment options:

1. Eyelid hygiene: Regular and proper eyelid hygiene is crucial in managing blepharitis. This includes:

- Warm compresses: Apply warm compresses to the closed eyelids for 5 to 10 minutes to loosen crusts and debris. This can help open up blocked oil glands and relieve symptoms.

- Lid scrubs: Use a gentle cleanser or mild baby shampoo diluted in warm water to clean the eyelids and lashes. Gently rub the solution along the lash line with clean fingertips or a clean cotton swab. Rinse thoroughly and pat dry.

- Lid massage: After cleaning the eyelids, gently massage the eyelid margins using clean fingertips. This can help express and clear blocked oil glands.

2. Topical antibiotics: In cases of blepharitis associated with bacterial overgrowth, your eye care professional may prescribe antibiotic ointments or drops to help control the infection and reduce inflammation. Follow the prescribed dosage and duration as instructed.

3. Steroid eye drops: In some cases of blepharitis with significant inflammation, short-term use of topical corticosteroid eye drops may be prescribed to reduce inflammation. However, long-term use of steroids is generally avoided due to potential side effects.

4. Artificial tears: Over-the-counter lubricating eye drops (artificial tears) can help alleviate dryness, discomfort, and irritation associated with blepharitis. They can be used as needed throughout the day.

5. Management of associated conditions: If blepharitis is associated with other conditions such as dry eyes or certain skin disorders, treating or managing these underlying conditions can help improve blepharitis symptoms. This may involve using additional treatments for dry eyes or addressing any skin conditions with appropriate medications.

6. Punctal plugs: In cases where dry eyes are a significant factor in blepharitis, your eye care professional may recommend the use of punctal plugs to conserve tears and increase moisture on the ocular surface.

It's important to follow the treatment plan recommended by your eye care professional and maintain good eyelid hygiene practices on a regular basis. If symptoms persist or worsen despite these measures, it's advisable to seek further evaluation and guidance from an eye care professional. They can assess your condition, identify any underlying factors, and recommend additional treatments or interventions as needed.

**Q6:**

I fell asleep with contact lenses two days ago and now I am suffering from painful red eye. What is the treatment?

**A6:**

I am not a medical professional, but I can offer some general information. If you fell asleep with contact lenses and are now experiencing a painful red eye, it could be due to a condition known as contact lens-related acute red eye or CLARE. However, several other issues could also cause similar symptoms. Regardless, it's essential to seek immediate medical attention from an eye doctor or healthcare professional for a proper diagnosis and treatment.

Some potential causes of painful red eyes after sleeping with contact lenses include:

1. Corneal Abrasion or Ulcer: Sleeping with contact lenses can cause dryness and increase the risk of corneal abrasions or ulcers, which can lead to severe pain and redness.

2. Bacterial or Fungal Infection: Prolonged contact lens wear, especially when sleeping with them, can create an environment for bacterial or fungal growth on the lenses or the eye's surface.

3. Contact Lens Overwear: Extended wear of contact lenses, especially without removing them for sleep, can irritate the cornea and cause redness and discomfort.

4. Giant Papillary Conjunctivitis (GPC): This is an allergic reaction that can develop due to the presence of contact lenses, causing redness, itching, and discomfort.

5. Contact Lens-Induced Keratitis (CLIK): This condition is characterized by inflammation of the cornea due to contact lens wear.

Treatment for painful red eye caused by contact lens-related issues will depend on the specific diagnosis. In general, you should:

1. Remove Contact Lenses: Take out the contact lenses immediately and do not wear them until you have consulted with an eye doctor.

2. Avoid Rubbing the Eyes: Rubbing the eyes can worsen the condition and lead to further irritation.

3. Use Lubricating Eye Drops: Artificial tears or lubricating eye drops may help soothe the eyes temporarily, but they won't treat the underlying cause.

4. Avoid Wearing Contact Lenses: Refrain from wearing contact lenses until you have seen an eye doctor and received appropriate treatment and clearance to resume wearing them.

5. Seek Medical Attention: Visit an eye doctor or healthcare professional for a thorough examination and proper diagnosis. They can provide you with the most suitable treatment plan based on your specific condition.

Remember, self-diagnosis and self-treatment are not advisable when it comes to eye-related issues. Prompt and professional evaluation is essential to prevent potential complications and promote the best possible outcome.

**Q7:**

What is the best timing for cataract surgery?

**A7:**

The timing of cataract surgery depends on several factors, including the severity of cataracts, the impact on visual function and quality of life, and the presence of any other eye conditions. The decision for cataract surgery is typically made in consultation with an ophthalmologist who evaluates these factors and recommends the most appropriate timing for each individual case. Here are some general considerations regarding the timing of cataract surgery:

1. Visual symptoms: If cataracts are causing significant visual symptoms that interfere with daily activities, such as difficulty reading, driving, or recognizing faces, cataract surgery may be considered. The goal is to improve visual function and quality of life.

2. Functional impairment: If cataracts significantly affect a person's ability to perform essential tasks, such as work-related activities or hobbies, surgery may be recommended to restore visual function and independence.

3. Safety concerns: In some cases, cataracts can increase the risk of accidents or falls, especially in low-light conditions. If compromised vision poses safety risks, cataract surgery may be advised.

4. Impact on other eye conditions: In individuals with other eye conditions, such as glaucoma or age-related macular degeneration, the timing of cataract surgery may be influenced by the need to optimize overall visual outcomes. Sometimes, simultaneous or staged procedures are planned to address multiple eye conditions.

5. Patient preferences and lifestyle: Patient preferences and lifestyle factors also play a role in determining the timing of cataract surgery. Some individuals may choose to undergo surgery earlier to improve their quality of life, while others may opt for a delayed approach.

It's important to note that cataract surgery is generally a safe and effective procedure with a high success rate. The ophthalmologist will carefully evaluate your individual circumstances and provide personalized recommendations regarding the timing of surgery. Regular eye examinations and discussions with your eye care professional can help monitor cataract progression and determine the optimal timing for intervention.

**Q8:**

What are the warning signs and symptoms for retinal detachment?

**A8:**

Retinal detachment is a serious condition that requires immediate medical attention. The following are common warning signs and symptoms that may indicate a retinal detachment:

1. Floaters: Sudden onset of an increase in the number or size of floaters—small specks or cobweb-like shapes that float across your field of vision. They may appear as dark spots or lines and may move when you try to focus on them.

2. Flashes of light: Perceiving flashes of light or seeing sudden bursts of light in your peripheral vision, even when there is no actual light source.

3. Shadow or curtain: The sensation of a shadow or curtain descending over a portion of your visual field. It may start from one side and progress gradually or suddenly.

4. Blurred or distorted vision: A sudden or gradual decrease in central or peripheral vision, which may cause objects to appear blurry or distorted.

5. Loss of peripheral vision: A significant loss of side vision or the sensation of a tunnel-like vision.

6. Pain or discomfort: Although pain is not always present, some individuals may experience eye pain, a feeling of pressure, or a general discomfort in the affected eye.

It's important to note that the presence of these signs and symptoms does not necessarily mean you have a retinal detachment, but they should not be ignored. If you experience any of these warning signs, it is crucial to seek immediate medical attention from an ophthalmologist or an emergency department. Prompt diagnosis and treatment are essential to increase the chances of successfully repairing a retinal detachment and preserving vision.

**Q9:**

What is the reason for excessive tearing and what is the treatment?

**A9:**

Excessive tearing, also known as epiphora, occurs when there is an overflow of tears from the eyes. This can happen due to various reasons, and it's essential to consult with an eye doctor or a healthcare professional to determine the underlying cause and provide appropriate treatment. Some common causes of excessive tearing include:

1. Eye Irritation: Foreign objects, dust, or allergens can irritate the eyes, leading to excessive tearing as a protective mechanism.

2. Blocked Tear Ducts: The tear drainage system can become blocked or narrowed, causing tears to accumulate and overflow.

3. Dry Eyes: Paradoxically, dry eyes can cause excessive tearing as the eyes try to compensate for the lack of sufficient lubrication.

4. Allergies: Allergic reactions to pollen, pet dander, or other allergens can trigger excessive tearing.

5. Conjunctivitis: Inflammation of the conjunctiva (pink eye) can cause watery eyes.

6. Eyelid Problems: Malposition of the eyelids, such as ectropion or entropion, can lead to improper tear drainage.

7. Infections or Injuries: Infections or injuries to the eye can cause excessive tearing.

The treatment for excessive tearing depends on the underlying cause:

- Eye drops or ointments may be prescribed to relieve eye irritation or dryness.

- Warm compresses and eyelid massage can help open blocked tear ducts.

- In some cases, minor surgical procedures may be required to correct eyelid malpositions or clear blocked tear ducts.

- Treating underlying allergies or infections can help reduce tearing.

- In situations where excessive tearing is due to dry eyes, artificial tears or other lubricating eye drops may be recommended.

It's essential to consult a healthcare professional or an eye doctor for a thorough examination and proper diagnosis. They will be able to identify the reason for your excessive tearing and provide appropriate treatment tailored to your specific condition.

**Q10:**

I have double vision, what are the causes and what is the treatment?

**A10:**

Experiencing double vision, also known as diplopia, can be concerning and may be a symptom of various underlying conditions. It's essential to seek immediate medical attention from an eye doctor or a healthcare professional for a comprehensive evaluation and accurate diagnosis. Some potential causes of double vision include:

1. Refractive Errors: Uncorrected nearsightedness, farsightedness, or astigmatism can lead to double vision, which is usually relieved when the refractive error is corrected with glasses or contact lenses.

2. Strabismus: Misalignment of the eyes can cause double vision, as the eyes are not properly aligned to focus on the same point.

3. Eye Muscle Weakness: Weak or paralyzed eye muscles can result in double vision, as the eyes may not move in sync.

4. Neurological Conditions: Various neurological problems, such as multiple sclerosis, stroke, brain tumors, or nerve palsies, can affect the control of eye movements and cause double vision.

5. Cataracts: A clouding of the lens in the eye can create double vision in the affected eye.

6. Diabetes: Diabetic neuropathy can affect the nerves that control eye movements and lead to double vision.

Treatment for double vision depends on the underlying cause:

- Refractive errors can often be corrected with glasses or contact lenses.

- Strabismus may require eye exercises, prism glasses, or in some cases, surgery to align the eyes properly.

- Neurological causes require specific treatment targeted at the underlying condition.

- Eye muscle weakness may be managed with vision therapy or, in some cases, surgery to strengthen or reposition the affected muscles.

- Cataracts can be treated with surgery to replace the clouded lens.

- In cases of double vision due to diabetes or other systemic conditions, managing the underlying health issue may help improve symptoms.

It's crucial to get a prompt and thorough examination by a qualified healthcare professional to determine the cause of your double vision and the most appropriate course of treatment. Ignoring double vision or self-diagnosing could lead to delayed treatment and potentially worsen the condition.

**Q11:**

What is the treatment for uveitis?

**A11:**

The treatment for uveitis, which is inflammation of the uvea (the middle layer of the eye), typically depends on the underlying cause and severity of the condition. The primary goal of treatment is to reduce inflammation, relieve symptoms, and prevent complications. Treatment options for uveitis may include:

1. Medications:

- Corticosteroids: These are often prescribed as eye drops, injections around the eye, or oral tablets to reduce inflammation.

- Immunosuppressive drugs: In more severe cases or when corticosteroids are not effective, medications that suppress the immune system may be prescribed. These include drugs such as methotrexate, azathioprine, mycophenolate mofetil, or cyclosporine.

- Biologic agents: In certain cases, biologic drugs such as adalimumab or infliximab may be used to target specific molecules involved in inflammation.

2. Local therapy:

- Eye drops: These may include corticosteroid eye drops or dilating eye drops to relieve pain and inflammation.

- Eye injections: In some cases, medication may be injected into the eye to directly target the inflammation.

3. Additional treatments:

- Cycloplegic agents: These medications help relax the muscles inside the eye, reduce pain, and prevent the formation of adhesions.

- Supportive therapy: Sunglasses or dark glasses can be helpful in reducing sensitivity to light (photophobia).

It's important to note that the specific treatment plan will depend on factors such as the type and location of uveitis, the presence of complications, and individual patient factors. Treatment should be determined by an ophthalmologist or a uveitis specialist who can tailor the approach to the individual's needs.

**Q12:**

I have bulging eyes, what is the cause and how can I treat it?

**A12:**

Bulging eyes, also known as proptosis or exophthalmos, is a condition where one or both eyes protrude from the eye sockets more than normal. It can be a symptom of various underlying conditions, and it's essential to seek medical attention from an eye doctor or a healthcare professional for proper evaluation and diagnosis. Some potential causes of bulging eyes include:

1. Thyroid Eye Disease (Graves' Disease): This is one of the most common causes of bulging eyes. It is an autoimmune condition where the immune system attacks the thyroid gland and the tissues around the eyes, causing inflammation and swelling.

2. Orbital Cellulitis: A severe infection of the tissues around the eye can lead to proptosis.

3. Eye Tumors: Tumors in or around the eye can cause the eyes to bulge.

4. Trauma: Injury or trauma to the eye or surrounding structures can result in bulging eyes.

5. Cavernous Sinus Fistula: An abnormal connection between blood vessels near the eye and the cavernous sinus can cause proptosis.

6. Orbital Inflammatory Disease: Inflammation of the tissues around the eye can lead to bulging.

7. Orbital Varices: Abnormal dilation of the veins around the eye can cause proptosis.

Treatment for bulging eyes depends on the underlying cause:

- For Graves' disease, treatment may involve managing the thyroid condition with medications, radioactive iodine therapy, or in some cases, surgery to reduce eye bulging.

- Infections or inflammations may require antibiotics or anti-inflammatory medications.

- Tumors may necessitate surgical intervention, radiation therapy, or other specialized treatments.

- Trauma-related proptosis may need immediate medical attention and possible surgical repair.

- Other causes will be managed based on the specific diagnosis.

It's crucial to see a healthcare professional promptly if you experience bulging eyes, as it could be a sign of a potentially serious medical condition. They will conduct a thorough evaluation, which may include imaging tests and blood work, to determine the cause and develop an appropriate treatment plan. Early diagnosis and intervention can help manage the underlying condition and prevent complications related to bulging eyes.

**Q13:**

What causes eyelid swelling?

**A13:**

Eyelid swelling can occur due to various reasons, including:

1. Blepharitis: This is an inflammation of the eyelid margins, often caused by bacterial infection, skin conditions (such as seborrheic dermatitis), or poor eyelid hygiene. It can lead to redness, swelling, itching, and crusting of the eyelids.

2. Chalazion: A chalazion is a blocked oil gland in the eyelid. It usually appears as a painless, firm lump or swelling on the eyelid. Over time, it can cause eyelid swelling, redness, and tenderness.

3. Sty (Hordeolum): A sty is an infection of the eyelid glands, usually caused by bacteria (Staphylococcus aureus). It appears as a tender, red bump on the eyelid, often with localized swelling.

4. Allergic reactions: Allergies to substances such as pollen, dust mites, pet dander, or certain medications can cause eyelid swelling, redness, itching, and watering of the eyes.

5. Contact dermatitis: Contact with irritating or allergenic substances, such as cosmetics, soaps, or metals (like nickel), can cause an allergic reaction on the eyelids, resulting in swelling, redness, and itching.

6. Orbital cellulitis: This is a serious infection of the tissues surrounding the eye, often originating from a sinus infection or trauma. Orbital cellulitis can cause eyelid swelling, redness, pain, fever, and decreased eye movement.

7. Preseptal cellulitis: Preseptal cellulitis is a less severe infection of the tissues in front of the eye, usually caused by bacteria. It leads to eyelid swelling, redness, and tenderness, but does not affect the actual eyeball or vision.

8. Trauma or injury: Any injury or trauma to the eye or eyelid, such as a blow to the face, can result in eyelid swelling.

9. Systemic conditions: Certain systemic conditions, such as thyroid eye disease, kidney disease, or heart failure, can cause fluid retention and lead to eyelid swelling.

These are some common causes of eyelid swelling. It's important to consult with a healthcare professional, such as an ophthalmologist or dermatologist, for an accurate diagnosis and appropriate treatment based on your specific symptoms and medical history.

**Q14:**
What is the treatment for optic disc edema due to idiopathic intracranial hypertension?

**A14:**

Optic disc edema due to idiopathic intracranial hypertension (IIH), also known as papilledema, requires a comprehensive treatment approach to manage the underlying condition and preserve vision. The primary goal of treatment is to reduce intracranial pressure and prevent further damage to the optic nerve. The specific treatment options may include:

1. Weight loss and lifestyle modifications: Since IIH is often associated with obesity, weight loss through a combination of dietary changes and exercise is typically recommended. This can help reduce intracranial pressure and improve symptoms.

2. Medications:

- Diuretics: These medications help reduce fluid retention and lower intracranial pressure. Commonly prescribed diuretics include acetazolamide and furosemide.

- Migraine medications: Certain medications used to treat migraines, such as topiramate, can help lower intracranial pressure in some cases.

3. Optic nerve sheath fenestration: In some instances, a surgical procedure called optic nerve sheath fenestration may be performed. This involves creating a small window in the covering of the optic nerve to relieve pressure and improve fluid drainage.

4. Ventriculoperitoneal shunt: In severe cases or when other treatments are ineffective, a ventriculoperitoneal shunt may be considered. This surgical procedure involves placing a tube (shunt) to divert excess cerebrospinal fluid from the brain's ventricles to the abdominal cavity, thereby reducing intracranial pressure.

5. Regular monitoring and follow-up: Close monitoring of optic disc edema and visual function is essential to assess the effectiveness of treatment and detect any changes or complications. This may involve regular eye examinations, visual field tests, and imaging studies.

It's important to note that the treatment of optic disc edema due to IIH should be tailored to the individual's specific condition and may require a multidisciplinary approach involving ophthalmologists, neurologists, and other healthcare professionals. Prompt diagnosis and treatment are crucial to prevent vision loss and manage the underlying condition effectively.

**Q15:**

My right eye looks smaller than my left one, what is the cause and how can I treat it?

**A15:**

I am not a medical professional, but I can provide some general information. If you notice that your right eye appears smaller than your left eye, it could be due to a condition called eyelid ptosis. Eyelid ptosis is a drooping or sagging of the upper eyelid, which can make the affected eye appear smaller.

Ptosis can have various underlying causes, and it's important to consult with an eye doctor or a healthcare professional for a proper evaluation and diagnosis. Some potential causes of eyelid ptosis include:

1. Aging: As we age, the muscles that lift the eyelids may weaken, leading to drooping eyelids.

2. Congenital Ptosis: Some individuals are born with ptosis due to a malformation of the eyelid muscles.

3. Nerve Damage: Damage to the nerve that controls the eyelid muscles can cause ptosis.

4. Eye Surgery: Ptosis can sometimes occur as a complication of eye surgery.

5. Neurological Conditions: Certain neurological disorders can affect the nerves that control the eyelid muscles.

Treatment for eyelid ptosis will depend on the underlying cause and the severity of the condition. Some potential treatment options include:

1. Surgical Correction: In cases of significant or severe ptosis, surgery may be recommended to lift the eyelid and improve its position.

2. Ptosis Crutches: In some cases, special glasses with ptosis crutches (eyelid props) may be used to lift the drooping eyelid.

3. Treatment of Underlying Conditions: If ptosis is a symptom of an underlying medical condition or nerve damage, addressing the root cause may help improve the eyelid position.

It's essential to seek medical advice from an eye doctor or a healthcare professional to determine the cause of the eyelid ptosis and receive appropriate treatment recommendations. They can perform a comprehensive examination and recommend the best course of action based on your individual circumstances. Remember that self-diagnosis and self-treatment are not advisable when it comes to eye-related issues, as prompt professional evaluation can help prevent potential complications and achieve the best possible outcome.

**Q16:**

What is the treatment for vitreous hemorrhage?

**A16:**

Vitreous hemorrhage refers to bleeding into the vitreous gel, which fills the space between the lens and retina in the eye. The treatment for vitreous hemorrhage depends on the underlying cause and severity of the condition. Here are some common treatment approaches:

1. Observation and conservative management: In some cases, small vitreous hemorrhages may resolve on their own over time without any specific treatment. The doctor may recommend observation and regular follow-up examinations to monitor the progress and ensure the hemorrhage is not worsening.

2. Treating the underlying cause: Addressing and managing the underlying cause of the vitreous hemorrhage is crucial. Depending on the specific condition, treatment options may include:

- Diabetic retinopathy: For individuals with diabetic retinopathy, controlling blood sugar levels, managing hypertension, and possibly undergoing laser treatment or intravitreal injections of anti-vascular endothelial growth factor (VEGF) medications may be recommended.

- Retinal tears or detachment: Surgical intervention, such as vitrectomy or retinal laser photocoagulation, may be necessary to repair retinal tears or reattach the retina.

- Retinal vein occlusion: Treatment options may include anti-VEGF injections, laser photocoagulation, or intravitreal corticosteroid injections.

- Age-related macular degeneration: In cases of vitreous hemorrhage associated with wet age-related macular degeneration, treatment may involve intravitreal injections of anti-VEGF medications.

3. Vitrectomy: In cases where the vitreous hemorrhage is severe, causing significant vision loss, or not resolving on its own, a surgical procedure called vitrectomy may be performed. Vitrectomy involves removing the vitreous gel and replacing it with a clear solution or gas bubble. This allows the surgeon to access and address the bleeding source, repair any associated retinal tears or detachments, and improve visual outcomes.

The treatment approach for vitreous hemorrhage requires an accurate diagnosis and careful evaluation by an ophthalmologist or a retina specialist. They will assess the underlying cause, severity, and individual circumstances to determine the most appropriate treatment strategy. Prompt medical attention is crucial to prevent further complications and preserve vision.

**Q17:**

What are the contraindications for refractive surgery?

**A17:**

Refractive surgery, such as LASIK (Laser-Assisted In Situ Keratomileusis) or PRK (Photorefractive Keratectomy), is a popular procedure used to correct vision problems, particularly nearsightedness, farsightedness, and astigmatism. However, not everyone is a suitable candidate for refractive surgery due to certain contraindications. Some common contraindications include:

1. Age: Refractive surgery is generally not recommended for individuals under the age of 18-21, as the eyes are still developing and vision may change during this time. Stable vision for a minimum period is required to ensure optimal results.

2. Unstable refractive error: Candidates should have a stable refractive error for at least 12-24 months, meaning that their vision prescription has not changed significantly during that time. Unstable refractive errors may indicate ongoing eye changes or underlying eye conditions that need to be addressed before considering surgery.

3. Eye diseases or conditions: Certain eye diseases or conditions can make refractive surgery inappropriate or risky. These include:

- Keratoconus: A progressive thinning and bulging of the cornea.

- Glaucoma: An eye condition characterized by increased intraocular pressure.

- Cataracts: Clouding of the lens of the eye.

- Herpes simplex or other active eye infections.

- Severe dry eye syndrome: Insufficient tear production or poor tear quality.

- Corneal dystrophy: Inherited disorders affecting the cornea.

4. Pregnancy and breastfeeding: Hormonal changes during pregnancy and while breastfeeding can affect vision stability. It is generally recommended to postpone refractive surgery until hormonal levels have stabilized post-pregnancy and breastfeeding.

5. Systemic health conditions: Certain systemic health conditions may be contraindications for refractive surgery. These can include autoimmune diseases, uncontrolled diabetes, collagen vascular diseases, immunodeficiency disorders, and connective tissue disorders. Each individual's medical history and overall health should be evaluated to determine if they are suitable candidates.

6. Unrealistic expectations: Candidates should have realistic expectations about the outcomes and limitations of refractive surgery. A thorough pre-operative evaluation, including discussions with the surgeon, can help ensure that the individual understands the potential risks, benefits, and expected outcomes of the procedure.

It is important to consult with an experienced ophthalmologist or refractive surgeon who can evaluate your individual circumstances and determine if you are a suitable candidate for refractive surgery. They will consider your eye health, overall health, vision stability, and other factors to make an informed decision.

**Q18:**

What is the treatment for keratoconus?

**A18:**

Keratoconus is a progressive eye disorder that causes thinning and bulging of the cornea, resulting in a cone-shaped protrusion. The treatment for keratoconus depends on the severity of the condition and the individual's specific needs. Here are some common treatment options:

1. Eyeglasses or contact lenses: In the early stages of keratoconus, eyeglasses or soft contact lenses may be sufficient to correct vision. Specialized contact lenses, such as rigid gas permeable (RGP) lenses or scleral lenses, are often prescribed to improve vision and provide better corneal support by creating a smooth optical surface.

2. Corneal cross-linking (CXL): Corneal cross-linking is a procedure that aims to strengthen the cornea and halt the progression of keratoconus. It involves applying riboflavin (vitamin B2) eye drops to the cornea and then exposing it to ultraviolet (UV) light. This combination promotes the formation of new collagen cross-links, increasing corneal rigidity and stability.

3. Intrastromal corneal ring segments (ICRS): ICRS, also known as corneal inserts or corneal implants, are small, clear, crescent-shaped devices that are surgically inserted into the cornea to flatten the cone-shaped cornea and improve vision. This procedure can help correct mild to moderate keratoconus and reduce the need for contact lenses.

4. Topography-guided custom ablation: This laser procedure, known as photorefractive keratectomy (PRK) or laser-assisted subepithelial keratomileusis (LASEK), is used to reshape the cornea and correct vision in cases where other treatments are not sufficient. It uses advanced mapping technology to precisely remove tissue from the cornea, improving its shape and visual outcomes.

5. Corneal transplant: In advanced cases of keratoconus where vision is severely affected, a corneal transplant (keratoplasty) may be necessary. During a corneal transplant, the damaged cornea is replaced with a healthy donor cornea. Depending on the extent of corneal involvement, different types of corneal transplant procedures, such as penetrating keratoplasty or deep anterior lamellar keratoplasty, may be performed.

It's important to note that the treatment approach for keratoconus should be personalized based on the individual's specific condition and needs. Regular monitoring and follow-up with an ophthalmologist or cornea specialist are crucial to assess the progression of keratoconus and determine the most appropriate treatment options.

**Q19:**

I am suffering from pain and localized redness on the left white part of my left eye, what is the diagnosis and treatment?

**A19:**

I am not a medical professional, but I can provide some general information. If you are experiencing pain and localized redness on the white part of your left eye, which is the sclera, there could be several potential causes for these symptoms. However, it's essential to consult with an eye doctor or a healthcare professional for a proper diagnosis and treatment plan. Some possible causes include:

1. Subconjunctival Hemorrhage: This occurs when a tiny blood vessel breaks beneath the conjunctiva (the clear membrane covering the white part of the eye), causing localized redness. It is usually painless and resolves on its own over time.

2. Scleritis: Inflammation of the sclera can cause severe pain and localized redness on the white part of the eye. Scleritis can be associated with autoimmune conditions or other systemic diseases and requires medical attention.

3. Episcleritis: Similar to scleritis, episcleritis is inflammation of the episclera (the tissue between the conjunctiva and sclera). It tends to be less severe than scleritis and may resolve on its own or with treatment.

4. Corneal Abrasion: A scratch on the cornea (the clear front part of the eye) can cause pain and localized redness on the sclera.

5. Foreign Object: A foreign object trapped under the eyelid or on the surface of the eye can cause irritation, pain, and redness.

6. Eye Infection: An infection of the eye, such as bacterial or viral conjunctivitis, can cause redness and discomfort on the sclera.

7. Allergic Reaction: An allergic reaction to pollen, pet dander, or other allergens can lead to redness and irritation on the white part of the eye.

Treatment for your symptoms will depend on the underlying cause:

- For subconjunctival hemorrhage, no specific treatment is usually required, and it will resolve on its own.

- Conditions like scleritis and episcleritis may require prescription eye drops or other medications to reduce inflammation and manage the underlying cause.

- Corneal abrasions, foreign objects, and eye infections may require treatment with eye drops, ointments, or in some cases, oral medications.

- Allergic reactions can be managed with antihistamine eye drops or oral medications.

It's crucial to see a healthcare professional for an accurate diagnosis and appropriate treatment plan. They can examine your eye, identify the specific cause of your symptoms, and provide you with the most suitable recommendations based on your medical history and the severity of the condition. Ignoring eye symptoms can potentially lead to complications, so seeking prompt medical attention is essential.

**Q20:**

What is the treatment for amblyopia?

**A20:**
Amblyopia, commonly known as "lazy eye," is a condition characterized by reduced vision in one eye due to abnormal visual development during childhood. The treatment for amblyopia aims to improve vision in the affected eye and promote binocular vision. Here are some common treatment options:

1. Prescription eyeglasses or contact lenses: Corrective lenses may be prescribed to address refractive errors (nearsightedness, farsightedness, or astigmatism) in the amblyopic eye. Clear vision provided by the glasses or contact lenses encourages the use of the weaker eye.

2. Patching therapy: Patching therapy involves covering the stronger eye with an eye patch for a designated period each day. This forces the child to rely on and use the amblyopic eye, stimulating visual development. The patching duration can vary depending on the severity of amblyopia and the child's age, but it is typically prescribed for several hours a day or as directed by an eye care professional.

3. Atropine eye drops: Instead of patching, atropine eye drops may be used to temporarily blur the vision in the stronger eye, promoting the use of the amblyopic eye. The drops are typically applied to the stronger eye once a day.

4. Vision therapy: Vision therapy involves a series of customized eye exercises and activities designed to improve visual skills and strengthen the eye-brain connection. It can help enhance binocular vision, eye coordination, and focusing abilities, which are often affected in amblyopia.

5. Surgical intervention: In some cases, if amblyopia is caused by a structural problem, such as a cataract or strabismus (misalignment of the eyes), surgery may be necessary. Surgery aims to correct the underlying issue and may be followed by additional treatments to optimize visual outcomes.

It is important to note that treatment for amblyopia is most effective when initiated early during childhood, as the visual system is more responsive to intervention during this critical period. Regular monitoring and follow-up with an eye care professional, such as an ophthalmologist or optometrist, are necessary to assess progress and modify treatment as needed.

The specific treatment plan for amblyopia should be determined by an eye care professional based on the individual's age, the severity of amblyopia, and any associated factors.
