# Supplementary material for: The use of artificial intelligence based chat bots in ophthalmology triage
Source: Eye (Lond). 2024 Nov 26;39(4):785–9. doi: 10.1038/s41433-024-03488-1 (PMC11885819; doi:10.1038/s41433-024-03488-1)
Supplement: Supplementary file 2 — Q and A BARD [file 41433_2024_3488_MOESM2_ESM.docx]

**Bard – Answers to Common Questions in Ophthalmology**

**Q1:**

I have a red eye with discomfort and discharge, what is the diagnosis and treatment?

**A1:**

The most likely diagnosis for your red eye with discomfort and discharge is conjunctivitis, also known as pink eye. Conjunctivitis is an inflammation of the conjunctiva, the clear membrane that lines the inside of your eyelids and the white part of your eye. It can be caused by a virus, bacteria, or an allergic reaction.

Viral conjunctivitis is the most common type of conjunctivitis. It is usually spread through contact with an infected person's eye discharge or mucus. Viral conjunctivitis usually goes away on its own within a week or two. There is no specific treatment for viral conjunctivitis, but you can relieve your symptoms with cool compresses, artificial tears, and over-the-counter pain relievers.

Bacterial conjunctivitis is less common than viral conjunctivitis, but it can be more serious. It is usually spread through contact with an infected person's eye discharge or mucus. Bacterial conjunctivitis can be treated with antibiotic eye drops or ointment.

Allergic conjunctivitis is caused by an allergic reaction to something in the environment, such as pollen, dust, or animal dander. It is usually accompanied by other allergy symptoms, such as sneezing, runny nose, and itchy eyes. Allergic conjunctivitis can be treated with over-the-counter antihistamines or decongestants.

If you have a red eye with discomfort and discharge, it is important to see a doctor to get a diagnosis and treatment. The doctor will examine your eye and ask you about your symptoms. They may also take a sample of your eye discharge to test for bacteria or viruses.

The treatment for your red eye will depend on the cause. If you have viral conjunctivitis, there is no specific treatment, but your doctor may prescribe eye drops or ointment to relieve your symptoms. If you have bacterial conjunctivitis, your doctor will prescribe antibiotic eye drops or ointment. If you have allergic conjunctivitis, your doctor may prescribe antihistamines or decongestants.

In addition to treatment, there are some things you can do to help relieve your symptoms and prevent the spread of conjunctivitis:

- Wash your hands often with soap and water.
- Avoid touching your eyes.
- If you wear contact lenses, remove them and clean them according to the manufacturer's instructions.
- Do not share eye makeup.

With proper treatment, most cases of conjunctivitis go away within a week or two. However, if your symptoms do not improve or get worse, see your doctor again.

**Q2:**

I have a painful, localized swelling of the eyelid, what is the diagnosis and treatment?

**A2:**

The most likely diagnosis for your painful, localized swelling of the eyelid is a stye. A stye is a small, red, tender bump on the eyelid that is caused by an infection of an oil gland in the eyelid. It is often accompanied by a feeling of fullness in the eyelid and a discharge of pus.

Styes are usually not serious and go away on their own within a few days or weeks. However, there are some things you can do to help relieve the pain and swelling:

- Apply a warm compress to the eyelid for 10-15 minutes several times a day.
- Use over-the-counter pain relievers, such as ibuprofen or acetaminophen.
- Wash your hands often with soap and water to prevent the spread of infection.

If your stye does not go away on its own or if it becomes worse, see your doctor. They may prescribe antibiotic eye drops or ointment to help clear up the infection.

In some cases, a stye can become a chalazion. A chalazion is a small, hard lump on the eyelid that is caused by a blockage of an oil gland. Chalazions are usually not painful and go away on their own within a few weeks or months. However, if a chalazion becomes large or bothersome, it can be removed by a doctor.

If you have a painful, localized swelling of the eyelid, it is important to see a doctor to get a diagnosis and treatment. The doctor will examine your eye and ask you about your symptoms. They may also prescribe antibiotics or other medications to help clear up the infection.

**Q3:**

I have a painless hemorrhage on the white part of my right eye, what is the diagnosis and treatment?

**A3:**

The most likely diagnosis for your painless hemorrhage on the white part of your right eye is a subconjunctival hemorrhage. A subconjunctival hemorrhage is a break in a blood vessel in the white part of the eye (sclera). This causes blood to leak into the space between the sclera and the conjunctiva, the thin, clear membrane that covers the sclera.

Subconjunctival hemorrhages are usually painless and do not affect vision. They are often caused by a sudden increase in blood pressure, such as from coughing, sneezing, or vomiting. They can also be caused by eye rubbing, injuries to the eye, or certain medications.

In most cases, subconjunctival hemorrhages go away on their own within a few weeks. There is no treatment needed. However, if you have a large subconjunctival hemorrhage or if it is accompanied by pain or vision changes, see an eye doctor.

Here are some things you can do to help the hemorrhage heal:

- Avoid rubbing your eyes.
- Use artificial tears to keep your eyes moist.
- Avoid wearing contact lenses until the hemorrhage has healed.

If you have any concerns about your subconjunctival hemorrhage, see an eye doctor. They can examine your eye and make sure that there is no underlying problem.

**Q4:**

What is the treatment for a retinal tear?

**A4:**

A retinal tear is a small hole or tear in the retina, the light-sensitive tissue at the back of the eye. If left untreated, a retinal tear can lead to a retinal detachment, which is a medical emergency that can cause permanent vision loss.

The treatment for a retinal tear depends on the size and location of the tear. Small tears that are not at risk of detachment can be monitored by an eye doctor. However, larger tears or tears that are at risk of detachment need to be treated to prevent further vision loss.

There are two main types of treatment for retinal tears:

- Laser photocoagulation: This treatment uses a laser to create small burns around the tear. The burns create scar tissue that helps to seal the tear and prevent it from detaching.
- Cryopexy: This treatment uses a freezing probe to create a scar around the tear. The scar tissue helps to seal the tear and prevent it from detaching.

In some cases, a retinal tear may require surgery to repair. The type of surgery that is performed will depend on the size and location of the tear.

If you have been diagnosed with a retinal tear, it is important to see an eye doctor right away to discuss treatment options. Early treatment can help to prevent a retinal detachment and preserve your vision.

Here are some additional information about retinal tears:

- Retinal tears are most common in people over the age of 50.
- Other risk factors for retinal tears include:
  - High myopia (nearsightedness)
  - Previous eye surgery
  - Injury to the eye
  - Certain medical conditions, such as diabetes and high blood pressure
- Symptoms of a retinal tear may include:
  - Flashes of light
  - Floaters
  - A curtain or shadow over part of your vision
  - Pain in the eye

If you experience any of these symptoms, it is important to see an eye doctor right away. Early diagnosis and treatment of retinal tears can help to prevent permanent vision loss.

**Q5:**

What is the treatment for blepharitis?

**A5:**

Blepharitis is an inflammation of the eyelids. It is often caused by a build-up of bacteria or oil on the eyelids. Blepharitis can cause a number of symptoms, including:

- Red, swollen eyelids
- Irritation and burning
- Eyelash loss
- Crusts on the eyelids
- Dry eyes
- Sensitivity to light

There is no cure for blepharitis, but it can be managed with treatment. The goal of treatment is to reduce inflammation and prevent the build-up of bacteria or oil on the eyelids.

There are a number of different treatments for blepharitis, including:

- Eyelid hygiene: This is the most important treatment for blepharitis. It involves cleaning the eyelids with warm water and a mild soap or cleanser. You can also use a cotton swab dipped in baby shampoo to clean your eyelids.
- Antibiotics: If blepharitis is caused by bacteria, antibiotics may be prescribed. Antibiotics can be taken orally or applied to the eyelids as eye drops or ointment.
- Steroids: Steroids can help to reduce inflammation. However, steroids should only be used for a short period of time, as they can have side effects, such as increased eye pressure.
- Other treatments: Other treatments for blepharitis include:
  - Artificial tears: Artificial tears can help to keep the eyes moist and prevent dryness.
  - Warm compresses: Warm compresses can help to reduce inflammation and loosen crusts on the eyelids.
  - Tar shampoo: Tar shampoo can help to reduce oil production on the eyelids.

If you have blepharitis, it is important to see an eye doctor to get a diagnosis and treatment plan. Treatment can help to relieve symptoms and prevent complications.

Here are some tips to help prevent blepharitis:

- Clean your eyelids regularly: This is the most important thing you can do to prevent blepharitis. Use warm water and a mild soap or cleanser, or a cotton swab dipped in baby shampoo.
- Avoid rubbing your eyes: Rubbing your eyes can irritate the eyelids and make blepharitis worse.
- Wear clean glasses or contact lenses: If you wear glasses or contact lenses, make sure to clean them regularly. This will help to prevent bacteria from building up on the lenses and causing blepharitis.
- Manage stress: Stress can make blepharitis worse. Find healthy ways to manage stress, such as exercise, yoga, or meditation.

If you have any concerns about blepharitis, see an eye doctor. They can diagnose the condition and recommend treatment.

**Q6:**

I fell asleep with contact lenses two days ago and now I am suffering from painful red eye. What is the treatment?

**A6:**

Sleeping in contact lenses can cause a number of problems, including:

- Corneal abrasion: This is a scratch on the cornea, the clear front of the eye. Corneal abrasions can be painful and can lead to infection.
- Infection: Sleeping in contact lenses can increase the risk of infection, such as keratitis or conjunctivitis.
- Discomfort: Sleeping in contact lenses can cause discomfort, such as redness, irritation, and dryness.

If you have a painful red eye after sleeping in contact lenses, it is important to see an eye doctor right away. The doctor will examine your eye and determine the cause of the pain. If you have a corneal abrasion, the doctor may prescribe antibiotic eye drops or ointment. If you have an infection, the doctor may prescribe antibiotics.

In the meantime, there are a few things you can do to help relieve the pain and discomfort:

- Remove your contact lenses: This is the most important thing you can do. Contact lenses can trap bacteria and irritate your eye.
- Use artificial tears: Artificial tears can help to keep your eye moist and relieve dryness.
- Apply a cold compress: A cold compress can help to reduce inflammation and pain.
- Avoid rubbing your eye: Rubbing your eye can further irritate the cornea.

If the pain and discomfort do not improve after a few days, or if you develop any other symptoms, such as light sensitivity or blurry vision, see an eye doctor again.

Here are some tips to help prevent sleeping in your contact lenses:

- Set an alarm: Set an alarm to remind yourself to take out your contact lenses before you go to bed.
- Use the right type of contact lenses: If you wear extended wear contact lenses, make sure to follow the instructions for wear time.
- Take out your contact lenses if you are feeling tired: If you are feeling tired, take out your contact lenses even if you are not going to bed right away.
- Clean your contact lenses regularly: Make sure to clean your contact lenses according to the manufacturer's instructions.

By following these tips, you can help to prevent the problems that can occur from sleeping in contact lenses.

**Q7:**

What is the best timing for cataract surgery?

**A7:**

The best timing for cataract surgery is when the cataracts are causing significant problems with your vision and are interfering with your daily activities. Some of the signs and symptoms of cataracts that may indicate it is time for surgery include:

- Blurry vision
- Increased sensitivity to light
- Halos around lights
- Double vision
- Difficulty driving at night
- Difficulty reading

There is no one-size-fits-all answer to the question of when is the best time for cataract surgery, as the timing will vary depending on the individual's symptoms and lifestyle. However, in general, cataract surgery is considered to be a safe and effective procedure, and it can significantly improve vision.

If you are considering cataract surgery, it is important to talk to your eye doctor about the best timing for you. They will be able to assess your individual situation and recommend the best course of treatment.

Here are some factors that may affect the timing of cataract surgery:

- The severity of your cataracts
- Your age and overall health
- Your lifestyle and activities
- Your preferences

Once you have decided that cataract surgery is right for you, your eye doctor will schedule the surgery. Cataract surgery is usually done on an outpatient basis, and it typically takes about 30 minutes per eye.

After surgery, you will need to wear an eye shield for a few days. You may also experience some mild discomfort, such as blurry vision, light sensitivity, and tearing. However, these symptoms usually go away within a few weeks.

In most cases, cataract surgery is a very successful procedure, and it can significantly improve vision. However, there are some risks associated with surgery, such as infection, bleeding, and damage to the eye.

If you are considering cataract surgery, it is important to talk to your eye doctor about the risks and benefits of the procedure. They can help you decide if cataract surgery is the right choice for you.

**Q8:**

What are the warning signs and symptoms for retinal detachment?

**A8:**

Retinal detachment is a serious eye condition that occurs when the retina, the light-sensitive tissue at the back of the eye, pulls away from the underlying tissue. Retinal detachment can cause sudden, severe vision loss.

The warning signs and symptoms of retinal detachment can include:

- Flashes of light: These are caused by the retina pulling away from the underlying tissue and can be seen as sudden, bright flashes of light.
- Floaters: These are small, dark spots or specks that float across your vision. They are caused by clumps of cells or protein in the vitreous, the clear gel that fills the inside of the eye.
- A curtain or shadow over part of your vision: This is a more serious symptom of retinal detachment and can indicate that the retina is detaching.
- Sudden, severe vision loss: This is the most serious symptom of retinal detachment and requires immediate medical attention.

If you experience any of these symptoms, it is important to see an eye doctor right away. Early diagnosis and treatment of retinal detachment can help to prevent permanent vision loss.

Here are some risk factors for retinal detachment:

- Age: Retinal detachment is more common in people over the age of 50.
- Myopia: People with myopia (nearsightedness) are more likely to develop retinal detachment.
- Previous eye surgery: People who have had previous eye surgery, such as cataract surgery, are at an increased risk of retinal detachment.
- Family history: People who have a family history of retinal detachment are more likely to develop the condition.
- Eye trauma: Eye trauma, such as a blow to the eye, can increase the risk of retinal detachment.

If you have any of these risk factors, it is important to see an eye doctor for regular eye exams. This will help to detect any changes in your retina and allow for early treatment if necessary.

**Q9:**

What is the reason for excessive tearing and what is the treatment?

**A9:**

Excessive tearing, also known as epiphora, is a condition that occurs when there is an imbalance between tear production and tear drainage. This can cause tears to overflow from the eye and run down the cheek.

There are many different causes of excessive tearing, including:

- Blocked tear ducts: The tear ducts are the small tubes that drain tears from the eye into the nose. If the tear ducts are blocked, tears can build up in the eye and cause excessive tearing.
- Inflammation: Inflammation of the eyelids or the eye itself can cause excessive tearing.
- Allergies: Allergies can cause the eyes to produce more tears.
- Foreign objects in the eye: If there is a foreign object in the eye, it can irritate the eye and cause excessive tearing.
- Dry eye syndrome: Dry eye syndrome is a condition that occurs when the eyes do not produce enough tears. This can cause the eyes to feel dry, irritated, and red.
- Eyelid problems: Problems with the eyelids, such as droopy eyelids or inward-turned eyelashes, can also cause excessive tearing.

The treatment for excessive tearing will depend on the underlying cause. If the tear ducts are blocked, they may need to be opened surgically. If inflammation is the cause, medications may be prescribed to reduce inflammation. If allergies are the cause, allergy medications may be prescribed. If there is a foreign object in the eye, it will need to be removed. If dry eye syndrome is the cause, artificial tears or other treatments may be prescribed.

If you have excessive tearing, it is important to see an eye doctor to determine the underlying cause and get appropriate treatment. Early diagnosis and treatment can help to prevent complications, such as corneal damage.

Here are some tips to help prevent excessive tearing:

- Avoid rubbing your eyes. Rubbing your eyes can irritate the eye and make excessive tearing worse.
- Use artificial tears. Artificial tears can help to keep the eyes moist and prevent dryness, which can contribute to excessive tearing.
- Wear sunglasses. Wearing sunglasses can help to protect your eyes from wind, dust, and other irritants that can cause excessive tearing.
- Manage allergies. If you have allergies, managing your allergies can help to reduce excessive tearing.
- Get regular eye exams. Regular eye exams can help to detect any problems with your eyes early on, so that they can be treated before they cause excessive tearing.

**Q10:**

I have double vision, what are the causes and what is the treatment?

**A10:**

Double vision, also known as diplopia, is a condition in which you see two images of a single object. It can be caused by a variety of factors, including:

- Problems with the muscles that control eye movement: These muscles work together to ensure that both eyes are pointing in the same direction. If one or more of these muscles is weak or paralyzed, it can cause double vision.
- Problems with the nerves that control eye movement: The nerves that control eye movement can be damaged by injury, disease, or tumor. This can also cause double vision.
- Problems with the brain: The brain is responsible for interpreting the images that the eyes see. If there is a problem with the brain, it can cause double vision.

The treatment for double vision will depend on the underlying cause. If the problem is caused by a weak or paralyzed muscle, surgery may be necessary to correct the muscle's alignment. If the problem is caused by a nerve problem, medications or surgery may be necessary to treat the underlying condition. If the problem is caused by a brain problem, treatment will depend on the underlying condition.

In some cases, double vision can be corrected with glasses or contact lenses. However, if the double vision is caused by a more serious underlying condition, such as a brain tumor, treatment may be more complex.

If you are experiencing double vision, it is important to see an eye doctor to determine the underlying cause and get appropriate treatment. Early diagnosis and treatment can help to prevent complications, such as falls or injuries.

Here are some tips to help prevent double vision:

- Get regular eye exams. Regular eye exams can help to detect any problems with your eyes early on, so that they can be treated before they cause double vision.
- Avoid activities that can put strain on your eyes. Activities such as reading in low light or using a computer for long periods of time can put strain on your eyes and make double vision worse.
- Manage stress. Stress can worsen double vision. Find healthy ways to manage stress, such as exercise, yoga, or meditation.

If you have any concerns about double vision, see an eye doctor. They can diagnose the condition and recommend treatment.

**Q11:**

What is the treatment for uveitis?

**A11:**

Uveitis is an inflammation of the uveal tract, which is the middle layer of the eye. It can affect any part of the uveal tract, including the iris (the colored part of the eye), the ciliary body (which produces the fluid that fills the inside of the eye), and the choroid (the layer of tissue behind the iris that helps to nourish the retina).

Uveitis can be caused by a variety of factors, including:

- Infection: Uveitis can be caused by bacteria, viruses, or fungi.
- Autoimmune disease: Uveitis can be a symptom of an autoimmune disease, such as ankylosing spondylitis or rheumatoid arthritis.
- Trauma: Uveitis can be caused by eye injury.
- Inflammatory bowel disease: Uveitis can be a symptom of inflammatory bowel disease, such as Crohn's disease or ulcerative colitis.
- Unknown: In some cases, the cause of uveitis is unknown.

The treatment for uveitis will depend on the underlying cause. If the uveitis is caused by an infection, antibiotics or antiviral medications may be prescribed. If the uveitis is caused by an autoimmune disease, medications to suppress the immune system may be prescribed. If the uveitis is caused by trauma, the treatment will depend on the severity of the injury.

In some cases, uveitis may go away on its own. However, in other cases, it may be a chronic condition that requires long-term treatment.

If you have uveitis, it is important to see an eye doctor to determine the underlying cause and get appropriate treatment. Early diagnosis and treatment can help to prevent complications, such as vision loss.

Here are some tips to help prevent uveitis:

- Get regular eye exams. Regular eye exams can help to detect any problems with your eyes early on, so that they can be treated before they cause uveitis.
- Avoid activities that can put strain on your eyes. Activities such as reading in low light or using a computer for long periods of time can put strain on your eyes and make uveitis worse.
- Manage stress. Stress can worsen uveitis. Find healthy ways to manage stress, such as exercise, yoga, or meditation.

If you have any concerns about uveitis, see an eye doctor. They can diagnose the condition and recommend treatment.

**Q12:**

I have bulging eyes, what is the cause and how can I treat it?

**A12:**

Bulging eyes, also known as exophthalmos, is a condition in which the eyes appear to protrude from the eye sockets. It can be caused by a variety of factors, including:

- Graves' disease: Graves' disease is an autoimmune disorder that causes the thyroid gland to produce too much thyroid hormone. This can lead to a number of symptoms, including bulging eyes.
- Thyroid eye disease: Thyroid eye disease is a condition that affects the tissues around the eye, including the muscles and fat. It can cause the eyes to bulge and can also lead to other symptoms, such as dry eyes, double vision, and eyelid retraction.
- Infection: Infections, such as sinus infections or orbital cellulitis, can cause inflammation around the eye, which can lead to bulging eyes.
- Trauma: Eye trauma, such as a blow to the eye, can cause the eye to bulge.
- Tumors: Tumors in the eye or around the eye can cause bulging eyes.

The treatment for bulging eyes will depend on the underlying cause. If the bulging eyes are caused by Graves' disease, treatment will involve medications to control the thyroid hormone levels. If the bulging eyes are caused by thyroid eye disease, treatment may involve medications, surgery, or a combination of both. If the bulging eyes are caused by an infection, antibiotics may be prescribed. If the bulging eyes are caused by trauma, the treatment will depend on the severity of the injury. If the bulging eyes are caused by a tumor, treatment will involve surgery or radiation therapy.

In some cases, bulging eyes may go away on their own. However, in other cases, it may be a chronic condition that requires long-term treatment.

If you have bulging eyes, it is important to see an eye doctor to determine the underlying cause and get appropriate treatment. Early diagnosis and treatment can help to prevent complications, such as vision loss.

Here are some tips to help prevent bulging eyes:

- Get regular eye exams. Regular eye exams can help to detect any problems with your eyes early on, so that they can be treated before they cause bulging eyes.
- Manage stress. Stress can worsen bulging eyes. Find healthy ways to manage stress, such as exercise, yoga, or meditation.

If you have any concerns about bulging eyes, see an eye doctor. They can diagnose the condition and recommend treatment.

**Q13:**

What causes eyelid swelling?

**A13:**

There are many causes of eyelid swelling. Some of the most common include:

- Infection: Eye infections, such as conjunctivitis or blepharitis, can cause eyelid swelling.
- Injury: Eye injuries, such as a blow to the eye, can also cause eyelid swelling.
- Allergies: Allergies can cause eyelid swelling, often along with other symptoms such as itching, redness, and tearing.
- Medications: Some medications, such as steroids, can cause eyelid swelling as a side effect.
- Hormonal changes: Hormonal changes, such as those that occur during pregnancy or menopause, can also cause eyelid swelling.
- Skin conditions: Skin conditions, such as eczema or psoriasis, can also cause eyelid swelling.
- Tumors: Tumors in or around the eye can also cause eyelid swelling.

If you have eyelid swelling, it is important to see an eye doctor to determine the underlying cause and get appropriate treatment. Early diagnosis and treatment can help to prevent complications, such as vision loss.

Here are some tips to help prevent eyelid swelling:

- Get regular eye exams. Regular eye exams can help to detect any problems with your eyes early on, so that they can be treated before they cause eyelid swelling.
- Avoid rubbing your eyes. Rubbing your eyes can irritate the eye and make eyelid swelling worse.
- Use artificial tears. Artificial tears can help to keep the eyes moist and prevent dryness, which can contribute to eyelid swelling.
- Manage allergies. If you have allergies, managing your allergies can help to reduce eyelid swelling.
- Wear sunglasses. Wearing sunglasses can help to protect your eyes from wind, dust, and other irritants that can cause eyelid swelling.

If you have any concerns about eyelid swelling, see an eye doctor. They can diagnose the condition and recommend treatment.

**Q14:**
What is the treatment for optic disc edema due to idiopathic intracranial hypertension?

**A14:**

Optic disc edema due to idiopathic intracranial hypertension (IIH) is a condition in which the optic nerve swells due to increased pressure inside the skull. This can lead to vision loss, and in some cases, blindness.

The treatment for optic disc edema due to IIH typically involves reducing the pressure inside the skull. This can be done with medications, such as acetazolamide or furosemide, or with surgery.

Medications: Acetazolamide is a diuretic that helps to reduce the amount of fluid in the body. Furosemide is another diuretic that can also be used to reduce fluid. In some cases, a combination of both medications may be used.

Surgery: If medications are not effective in reducing the pressure inside the skull, surgery may be necessary. There are a number of different surgical procedures that can be used to treat IIH, including:

- Venous sinus stenting: This procedure involves placing a stent in a vein that drains blood from the brain. This can help to improve the flow of blood and reduce the pressure inside the skull.
- Cerebellopontine angle shunt: This procedure involves placing a shunt in the brain that drains excess cerebrospinal fluid (CSF) into another part of the body. This can help to reduce the pressure inside the skull.

The treatment for optic disc edema due to IIH will vary depending on the severity of the condition and the individual's response to treatment. Early diagnosis and treatment are important to prevent vision loss.

Here are some tips to help prevent optic disc edema due to IIH:

- Get regular eye exams. Regular eye exams can help to detect any problems with your eyes early on, so that they can be treated before they cause optic disc edema.
- Lose weight. If you are overweight or obese, losing weight can help to reduce the pressure inside your skull.
- Avoid caffeine and alcohol. Caffeine and alcohol can dehydrate you, which can increase the pressure inside your skull.
- Manage stress. Stress can increase the pressure inside your skull. Find healthy ways to manage stress, such as exercise, yoga, or meditation.

If you have any concerns about optic disc edema, see an eye doctor. They can diagnose the condition and recommend treatment.

**Q15:**

My right eye looks smaller than my left one, what is the cause and how can I treat it?

**A15:**

There are a few possible reasons why your right eye might look smaller than your left one.

- Asymmetrical features: It's possible that your eyes are simply asymmetrical, which is perfectly normal. This means that one eye may be slightly larger or smaller than the other, but it doesn't mean that there's anything wrong with your vision.
- Amblyopia: Amblyopia, also known as lazy eye, is a condition that causes one eye to develop poorly. This can happen if the brain doesn't receive enough visual input from one eye, usually due to amblyopia. In some cases, amblyopia can cause one eye to appear smaller than the other.
- Ptosis: Ptosis is a condition that causes the eyelid to droop. This can happen due to a number of factors, including age, injury, or surgery. If the ptosis is severe, it can make one eye appear smaller than the other.
- Ocular tumor: A tumor in or around the eye can cause one eye to appear larger or smaller than the other. This is a serious condition that requires prompt medical attention.

If you're concerned about the size of your eyes, it's important to see an eye doctor to determine the cause. The doctor will perform a comprehensive eye exam and may order additional tests, such as an MRI or CT scan, to rule out any underlying medical conditions.

Depending on the cause of your asymmetrical eyes, there are a few possible treatments that may help. For example, if you have amblyopia, you may be prescribed eyeglasses or patching therapy to help improve vision in the affected eye. If you have ptosis, you may need surgery to lift the eyelid. And if you have an ocular tumor, you may need surgery to remove the tumor.

The treatment for asymmetrical eyes will vary depending on the underlying cause. Early diagnosis and treatment are important to prevent any further vision loss.

**Q16:**

What is the treatment for vitreous hemorrhage?

**A16:**

Vitreous hemorrhage is a condition in which blood leaks into the vitreous, the clear gel that fills the inside of the eye. This can cause a number of symptoms, including floaters, blurry vision, and flashes of light.

In most cases, vitreous hemorrhages will clear up on their own within a few weeks. However, if the hemorrhage is large or does not clear up on its own, treatment may be necessary.

The treatment for vitreous hemorrhage will depend on the size and location of the hemorrhage. In some cases, no treatment may be necessary. However, if the hemorrhage is large or does not clear up on its own, treatment may be necessary.

Some of the treatment options for vitreous hemorrhage include:

- Observation: If the hemorrhage is small and does not cause any significant symptoms, your doctor may recommend simply observing it. In most cases, the hemorrhage will clear up on its own within a few weeks.
- Laser treatment: Laser treatment can be used to seal off the blood vessels that are bleeding into the vitreous. This can help to stop the bleeding and prevent the hemorrhage from getting larger.
- Vitrectomy: Vitrectomy is a surgical procedure that involves removing the vitreous from the eye. This is a more invasive procedure, but it can be effective in clearing up large or persistent vitreous hemorrhages.

The treatment for vitreous hemorrhage will vary depending on the individual case. Your doctor will discuss the best treatment options for you based on the size and location of the hemorrhage, as well as your overall health.

Here are some tips to help prevent vitreous hemorrhage:

- Get regular eye exams. Regular eye exams can help to detect any problems with your eyes early on, so that they can be treated before they cause vitreous hemorrhage.
- Avoid activities that can put strain on your eyes. Activities such as reading in low light or using a computer for long periods of time can put strain on your eyes and make vitreous hemorrhage more likely.
- Manage your blood pressure. High blood pressure can increase your risk of vitreous hemorrhage. Managing your blood pressure can help to reduce your risk.

If you have any concerns about vitreous hemorrhage, see an eye doctor. They can diagnose the condition and recommend treatment.

**Q17:**

What are the contraindications for refractive surgery?

**A17:**

Refractive surgery is a type of eye surgery that can correct vision problems such as nearsightedness, farsightedness, and astigmatism. However, there are some conditions that can make refractive surgery a contraindication, meaning that it is not advisable or safe to undergo the procedure.

Some of the most common contraindications for refractive surgery include:

- Active eye infections: If you have an active eye infection, such as conjunctivitis or keratitis, refractive surgery should be postponed until the infection has cleared up.
- Dry eyes: If you have dry eyes, refractive surgery may make your dry eye symptoms worse.
- Astigmatism over 6 diopters: Refractive surgery is not as effective for astigmatism over 6 diopters.
- Thin corneas: If you have thin corneas, refractive surgery may be more risky.
- Preexisting retinal problems: If you have preexisting retinal problems, such as retinal detachment or macular degeneration, refractive surgery may make these problems worse.
- Pregnancy or breastfeeding: Refractive surgery is not recommended during pregnancy or breastfeeding.
- Unrealistic expectations: If you have unrealistic expectations about what refractive surgery can achieve, you may be disappointed with the results.

If you are considering refractive surgery, it is important to discuss your individual circumstances with your eye doctor. They can help you determine if refractive surgery is right for you and, if so, which type of procedure is best suited for your needs.

Here are some additional things to consider before undergoing refractive surgery:

- Your age: Refractive surgery is generally not recommended for people under the age of 18.
- Your lifestyle: If you are active in sports or other activities that put your eyes at risk, you may want to consider other options, such as glasses or contact lenses.
- Your budget: Refractive surgery can be expensive, so it is important to factor in the cost of the procedure and any potential complications.

If you have any questions or concerns about refractive surgery, be sure to talk to your eye doctor. They can help you make the best decision for your individual needs.

**Q18:**

What is the treatment for keratoconus?

**A18:**

Keratoconus is a progressive eye condition that causes the cornea to become thinner and bulge forward. This can lead to blurred vision, glare, and sensitivity to light.

There is no cure for keratoconus, but there are treatments that can help to slow the progression of the condition and improve vision.

The treatment for keratoconus will vary depending on the severity of the condition. In mild cases, glasses or contact lenses may be enough to correct vision. In more severe cases, surgery may be necessary.

Some of the treatment options for keratoconus include:

- Rigid gas permeable (RGP) contact lenses: RGP contact lenses are a type of hard contact lens that can help to improve vision in people with keratoconus.
- Scleral contact lenses: Scleral contact lenses are a type of large-diameter contact lens that can help to improve vision in people with keratoconus.
- Intracorneal ring segments (ICRS): ICRS are small, plastic rings that are implanted in the cornea to help reshape it. This can help to improve vision in people with keratoconus.
- Corneal crosslinking (CXL): CXL is a procedure that uses ultraviolet light and riboflavin to strengthen the cornea. This can help to slow the progression of keratoconus.
- Keratoprosthesis: A keratoprosthesis is a type of artificial cornea that can be implanted in people with advanced keratoconus. This can provide improved vision in people who have not responded to other treatments.

The treatment for keratoconus will vary depending on the individual case. Your eye doctor will discuss the best treatment options for you based on the severity of your condition and your overall health.

Here are some tips to help prevent keratoconus:

- Get regular eye exams. Regular eye exams can help to detect keratoconus early on, so that it can be treated before it causes vision loss.
- Avoid rubbing your eyes. Rubbing your eyes can irritate the cornea and make keratoconus worse.
- Wear sunglasses. Wearing sunglasses can help to protect your eyes from the sun's ultraviolet rays, which can damage the cornea.

If you have any concerns about keratoconus, see an eye doctor. They can diagnose the condition and recommend treatment.

**Q19:**

I am suffering from pain and localized redness on the left white part of my left eye, what is the diagnosis and treatment?

**A19:**

The pain and localized redness on the left white part of your left eye could be due to a number of different conditions, including:

- Conjunctivitis: Conjunctivitis is an inflammation of the conjunctiva, the thin, clear tissue that covers the white part of the eye and the inside of the eyelids. It is a common condition that can be caused by bacteria, viruses, or allergies.
- Blepharitis: Blepharitis is an inflammation of the eyelids. It can cause redness, swelling, and crusting of the eyelids.
- Keratoconjunctivitis sicca (KCS): KCS, also known as dry eye syndrome, is a condition that occurs when the eyes do not produce enough tears. This can cause dryness, irritation, and redness of the eyes.
- Pterygium: Pterygium is a growth of tissue on the white part of the eye. It is usually caused by exposure to ultraviolet (UV) rays.
- Subconjunctival hemorrhage: A subconjunctival hemorrhage is a blood clot that forms under the conjunctiva. It is usually caused by a minor injury to the eye.

It is important to see an eye doctor to get a diagnosis and treatment for the pain and localized redness on the left white part of your left eye. The doctor will examine your eye and ask you about your symptoms. They may also order tests, such as a fluorescein eye stain, to help diagnose the condition.

The treatment for the pain and localized redness on the left white part of your left eye will depend on the underlying condition. For example, if conjunctivitis is the cause, the doctor may prescribe antibiotics or antiviral medications. If blepharitis is the cause, the doctor may recommend eyelid hygiene measures, such as warm compresses and eyelid scrubs. If KCS is the cause, the doctor may recommend artificial tears or other treatments to increase tear production. If pterygium is the cause, the doctor may recommend surgery to remove the growth. If a subconjunctival hemorrhage is the cause, no treatment is usually necessary. The blood clot will usually go away on its own within a few weeks.

Here are some tips to help prevent these conditions:

- Get regular eye exams. Regular eye exams can help to detect these conditions early on, so that they can be treated before they cause damage to your eyes.
- Wear sunglasses. Wearing sunglasses can help to protect your eyes from UV rays, which can cause these conditions.
- Manage your allergies. If you have allergies, managing your allergies can help to prevent these conditions.
- Avoid rubbing your eyes. Rubbing your eyes can irritate your eyes and make these conditions worse.

If you have any concerns about the pain and localized redness on the left white part of your left eye, see an eye doctor. They can diagnose the condition and recommend treatment.

**Q20:**

What is the treatment for amblyopia?

**A20:**
Amblyopia, also known as lazy eye, is a condition in which the brain does not properly develop the vision in one eye. This can happen if the eye is not used as much as the other eye, usually due to a refractive error (such as nearsightedness, farsightedness, or astigmatism) or strabismus (crossed eyes).

The treatment for amblyopia depends on the age of the child and the severity of the condition. In children under the age of 7, the most common treatment is patching therapy. This involves covering the stronger eye with a patch for a certain number of hours each day. This forces the brain to use the weaker eye more, which can help to improve vision.

In older children and adults, other treatments may be used, such as vision therapy or surgery. Vision therapy involves exercises that help to improve the coordination between the eyes and the brain. Surgery may be used to correct the strabismus or to improve the alignment of the eyes.

The earlier amblyopia is treated, the more likely it is that vision can be improved. However, even adults with amblyopia can benefit from treatment.

Here are some tips to help prevent amblyopia:

- Get regular eye exams. Regular eye exams can help to detect amblyopia early on, so that it can be treated before it causes vision loss.
- Make sure your child's eyes are aligned properly. If your child's eyes are crossed, see an eye doctor to have them checked for strabismus.
- If your child has a refractive error, such as nearsightedness, farsightedness, or astigmatism, make sure they wear glasses or contact lenses to correct the error.
- Encourage your child to use both eyes equally. If your child has amblyopia, they may tend to favor one eye over the other. Encourage them to use both eyes equally, even if it is difficult at first.

If you have any concerns about amblyopia, see an eye doctor. They can diagnose the condition and recommend treatment.
