# Supplementary material for: The use of artificial intelligence based chat bots in ophthalmology triage
Source: Eye (Lond). 2024 Nov 26;39(4):785–9. doi: 10.1038/s41433-024-03488-1 (PMC11885819; doi:10.1038/s41433-024-03488-1)
Supplement: Supplementary file 3 — Supplementary Materials [file 41433_2024_3488_MOESM3_ESM.docx]

**Supplemental Materials:**

**Supplemental data 1** – Chat GPT's answers to common questions in ophthalmology

**Supplemental data 2** – BARD's answers to common questions in ophthalmology
